# Leveraging Multimodal Foundation Models in Biliary Tract Cancer Research

**DOI:** 10.3390/tomography11090096

**Published:** 2025-08-25

**Authors:** Yashbir Singh, Jesper B. Andersen, Quincy A. Hathaway, Diana V. Vera-Garcia, Varekan Keishing, Sudhakar K. Venkatesh, Sara Salehi, Davide Povero, Michael B. Wallace, Gregory J. Gores, Yujia Wei, Natally Horvat, Bradley J. Erickson, Emilio Quaia

**Affiliations:** 1 Radiology, Mayo Clinic, Rochester, MN 55905, USA; keishing.varekan@mayo.edu (V.K.); venkatesh.sudhakar@mayo.edu (S.K.V.); salehi.sara@mayo.edu (S.S.); yujia.wei@mayo.edu (Y.W.); horvat.natally@mayo.edu (N.H.); bje@mayo.edu (B.J.E.); 2 Biotech Research and Innovation Centre (BRIC), Department of Health and Medical Sciences, University of Copenhagen, DK-1165 Copenhagen, Denmark; jesper.andersen@bric.ku.dk; 3 Department of Radiology, University of Pennsylvania, Philadelphia, PA 19104, USA; quincy.hathaway@pennmedicine.upenn.edu; 4 Division of Gastroenterology & Hepatology, Mayo Clinic, Jacksonville, FL 32224, USA; veragarcia.diana@mayo.edu (D.V.V.-G.);; 5 Division of Gastroenterology & Hepatology, Mayo Clinic, Rochester, MN 55905, USA; povero.davide@mayo.edu (D.P.); gores.gregory@mayo.edu (G.J.G.); 6 Department of Radiology, Università di Padova, 35122 Padova, Italy; emilio.quaia@unipd.it

**Keywords:** biliary tract cancer, cholangiocarcinoma, artificial intelligence, multimodal foundation models, precision oncology, biomarkers, drug repurposing

## Abstract

This review explores how multimodal foundation models (MFMs) are transforming biliary tract cancer (BTC) research. BTCs are aggressive malignancies with poor prognosis, presenting unique challenges due to difficult diagnostic methods, molecular complexity, and rarity. Importantly, intrahepatic cholangiocarcinoma (iCCA), perihilar cholangiocarcinoma (pCCA), and distal bile duct cholangiocarcinoma (dCCA) represent fundamentally distinct clinical entities, with iCCA presenting as mass-forming lesions amenable to biopsy and targeted therapies, while pCCA manifests as infiltrative bile duct lesions with challenging diagnosis and primarily palliative management approaches. MFMs offer potential to advance research by integrating radiological images, histopathology, multi-omics profiles, and clinical data into unified computational frameworks, with applications tailored to these distinct BTC subtypes. Key applications include enhanced biomarker discovery that identifies previously unrecognizable cross-modal patterns, potential for improving currently limited diagnostic accuracy—though validation in BTC-specific cohorts remains essential—accelerated drug repurposing, and advanced patient stratification for personalized treatment. Despite promising results, challenges such as data scarcity, high computational demands, and clinical workflow integration remain to be addressed. Future research should focus on standardized data protocols, architectural innovations, and prospective validation studies. The integration of artificial intelligence (AI)-based methodologies offers new solutions for these historically challenging malignancies. However, current evidence for BTC-specific applications remains largely theoretical, with most studies limited to proof-of-concept designs or related cancer types. Comprehensive clinical validation studies and prospective trials demonstrating patient benefit are essential prerequisites for clinical implementation. The timeline for evidence-based clinical adoption likely extends 7–10 years, contingent on successful completion of validation studies addressing current evidence gaps.

## 1. Evidence Classification Framework

To provide clarity regarding the maturity of different MFM applications in BTC, we employ the following evidence hierarchy throughout this review:

Level 1: Validated in prospective, multi-center BTC clinical trials;

Level 2: Demonstrated in retrospective, Multi-Institutional BTC-specific cohorts with external validation;

Level 3: Proof-of-concept in single-institution BTC cohorts or biologically relevant cancers;

Level 4: Theoretical or in silico hypothesis requiring validation.

Each claim is marked with its corresponding evidence level (e.g., [Level 3]) to distinguish between validated findings and theoretical potential.

## 2. Important Clinical Considerations

Evidence Limitation Notice: While this review explores the potential applications of multimodal foundation models in BTC research, readers should note that:

1. No MFM approach has achieved prospective clinical validation in BTC-specific populations.
2. Most evidence derives from related cancer types or computational studies.
3. Cost-effectiveness and real-world implementation feasibility remain unproven.
4. Current applications should be considered investigational and require institutional review board approval.

Healthcare providers should not alter clinical practice based on the theoretical potential described in this review without appropriate validation studies and regulatory approval.

## 3. Introduction

Biliary tract cancers (BTCs) represent a compelling example of malignancies where therapeutic progress has lagged dramatically behind other cancer types. While 5-year survival rates for breast cancer have improved from 75% to 91% over the past three decades, and melanoma mortality has decreased by 7% annually following immunotherapy introduction, BTC survival remains stagnant below 20% across all subtypes [1,2,3]. This therapeutic stagnation, combined with rising global incidence and complex molecular heterogeneity, creates an urgent need for transformative computational approaches that can accelerate discovery and improve clinical outcomes. The genomic landscape of BTCs reveals distinct molecular alterations across anatomical subtypes, with iCCA often harboring *IDH1/2* mutations and *FGFR2* fusions, pCCA and dCCA showing *KRAS* mutations and *PRKACA/PRKACB* fusions, while gallbladder cancer (GBC) is often characterized by *ERBB* family amplifications [4].

Despite originating from the bile duct epithelium, iCCA and pCCA represent fundamentally different clinical entities that pose distinct challenges for diagnosis, treatment, and research [1,3] (Table 1). iCCA typically presents as a mass-forming lesion within the liver parenchyma, allowing for relatively straightforward tissue acquisition through core needle biopsy and molecular profiling [5,6]. This accessibility has enabled characterization of distinct driver mutations and led to targeted therapeutic approaches with demonstrable clinical benefit [7,8]. In stark contrast, pCCA manifests primarily as an infiltrative process at the biliary hilum, creating significant diagnostic challenges with conventional sampling techniques yielding sensitivity rates below 60% [9]. The therapeutic landscape also differs dramatically: while iCCA management follows paradigms similar to primary liver malignancies with opportunities for surgical resection and targeted therapies, pCCA management varies significantly by resectability status. For localized, resectable tumors, surgical resection with curative intent represents the standard approach, often requiring complex hepatectomy with biliary reconstruction. For unresectable disease or patients presenting with biliary obstruction, palliative interventions including stenting procedures become the primary focus, alongside systemic therapy considerations [3,10]. These profound differences necessitate distinct approaches to both clinical management and research applications of advanced computational methodologies, as will be discussed throughout this review.

Multimodal foundation models (MFMs) represent an emerging paradigm in artificial intelligence (AI), designed to integrate and process heterogeneous data types simultaneously [5]. These models build upon recent advances in deep learning architectures to analyze complex, multi-dimensional medical data.

Current diagnostic workflows for symptomatic patients involve integration of complex datasets including structured (laboratory values, genomic risk data) and unstructured (free text electronic health records) clinical information alongside multimodal imaging (MRI, MRCP, cholangiography, cholangioscopy). While these modalities are performed after clinical presentation, MFMs may enhance diagnostic accuracy and reduce time to definitive diagnosis in symptomatic patients. True early detection—identification of malignancy before symptom onset—remains a largely unmet need in BTC, as current screening approaches lack sufficient sensitivity and specificity for the predominant asymptomatic population. The integration of risk stratification models incorporating genetic predisposition, environmental factors, and subclinical biomarkers represents a potential future application, though evidence for such approaches remains theoretical.

**Figure 1:**
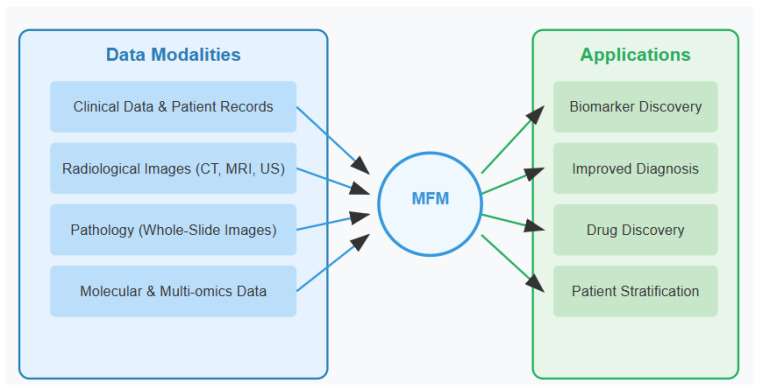
Multimodal foundation models (MFM) in biliary tract cancer (BTC) research.

Unlike traditional machine learning models that operate individually on single data modalities, MFMs can analyze text, images, genomic sequences, and clinical measurements within a unified computational framework. These models encompass various architectures including Transformers, GANs (Generative Adversarial Networks), encoder–decoder frameworks, and diffusion models, each offering unique strengths for multimodal integration. By leveraging self-supervised learning techniques on vast, diverse datasets, MFMs develop generalizable representations that transfer effectively across domains and tasks (Figure 1) [6]. Recent advances in transformer-based architectures have enabled unprecedented performance in cross-modal tasks, establishing MFMs as powerful tools for complex biomedical applications [13].

In clinical settings, MFMs have demonstrated promising results in various diseases. For instance, in hepatocellular carcinoma (HCC), multimodal models integrating radiological images with genomic and clinical data have improved early detection and prognostic accuracy [6]. Similarly, in neurodegenerative disorders, MFMs combining neuroimaging, cognitive assessments, and genetic markers have shown enhanced diagnostic precision and treatment response prediction [13].

This narrative review explores the intersection of MFMs and BTC research, highlighting both the clinical needs driving innovation and the technical considerations for AI implementation. For clinicians managing BTC patients, we provide a framework for understanding how these advanced AI systems can augment diagnostic workflows, treatment selection, and prognostic evaluation. For AI researchers and bioinformaticians, we offer insights into the unique challenges of BTC data and outline architectural considerations for developing clinically relevant models. By bridging these perspectives, we aim to accelerate the translation of cutting-edge AI methodologies into practical clinical tools for this challenging malignancy.

### 3.1. Distinct Clinical Entities: iCCA Versus pCCA

Biliary tract cancers represent a heterogeneous group of malignancies that exhibit markedly different characteristics based on anatomical location. While sharing a common tissue of origin in the bile duct epithelium, iCCA and pCCA present as fundamentally distinct clinical entities with significant implications for diagnosis, molecular characterization, and treatment approaches [1].

iCCA typically manifests as a mass-forming lesion within the liver parenchyma, making it relatively accessible for diagnostic tissue acquisition through core needle biopsy. This availability of adequate tissue samples facilitates comprehensive molecular profiling, revealing characteristic genetic alterations including *IDH1/2* mutations and *FGFR2* fusions that are largely unique to this anatomical subtype [4,5]. These distinctive molecular features have led to successful targeted therapeutic approaches, as evidenced by the development of FGFR inhibitors (pemigatinib, infigratinib) for FGFR2 fusion-positive iCCA and IDH inhibitors (ivosidenib) for IDH1-mutated iCCA [7,8]. Advanced diagnostic methods also include fluorescence in situ hybridization (FISH) of biliary brush cytology, as well as advanced optical intraductal imaging/cholangioscopy including machine learning-enhanced computer vision technologies [14]. The molecular landscape of pCCA is characterized by *KRAS* mutations and *PRKACA/PRKACB* fusions but has fewer clearly defined and clinically actionable alterations compared to iCCA [4]. The therapeutic approach also differs significantly between these subtypes. Management of iCCA follows paradigms similar to primary liver malignancies, with surgical resection as the primary curative option for localized disease, and consideration of locoregional therapies (ablation, transarterial chemoembolization) for selected patients [10]. Systemic therapy for advanced disease has evolved from the standard gemcitabine/cisplatin (GemCis) regimen to include durvalumab or pembrolizumab, improving outcomes based on the TOPAZ-1 and KEYNOTE-966 trials, respectively [15].

pCCA management, however, centers on addressing biliary obstruction and associated complications. Palliative biliary stenting often represents a critical intervention to relieve jaundice, prevent cholangitis, and improve quality of life [3]. The complex biliary anatomy at the hilum frequently necessitates multiple stent placement to adequately drain obstructed segments. Surgical approaches for pCCA are technically challenging and typically involve extensive hepatectomy with biliary and vascular reconstruction, limiting their application to highly selected patients at specialized centers [10]. These anatomical distinctions have profound implications for the application of multimodal foundation models (MFMs) in these diseases. For iCCA, MFMs can integrate radiological features of mass lesions with molecular profiles to identify novel patient subgroups with distinct therapeutic vulnerabilities. For pCCA, the value of MFMs may be greater in enhancing diagnostic accuracy through the integration of imaging features, cholangiography findings, cytology results, and limited molecular data to improve early detection in this diagnostically challenging entity [11,12]. By recognizing these fundamental differences between iCCA and pCCA throughout our discussion of multimodal approaches, we can better address the specific clinical challenges and opportunities each subtype presents.

### 3.2. Current State of Evidence in BTC-Specific MFM Applications

While this review explores the potential of MFMs in BTC research, it is crucial to acknowledge the current limitations in evidence. A systematic review of published literature reveals that only three studies have directly applied multimodal AI approaches specifically to BTC cohorts [Level 2–3], with most evidence extrapolated from related gastrointestinal malignancies or pan-cancer datasets with limited BTC representation. This evidence gap stems from the rarity of BTC (incidence 1–2 per 100,000) and the challenges in assembling sufficiently large, well-characterized cohorts for robust AI model development and validation.

### 3.3. Molecular Profiling and Biomarker Discovery

The molecular heterogeneity of BTCs presents significant challenges for implementing precision medicine approaches in clinical practice [16]. Current molecular profiling techniques typically analyze genomic alterations in isolation from morphological and clinical features, creating a fragmented view of the disease [Level 3]. The identification of actionable molecular alterations remains a critical unmet need, with fewer than 30% of BTC patients harboring targetable genomic alterations under current classification schemes [Level 3]. Conventional biomarker discovery approaches in BTCs have yielded limited clinical utility [Level 3]. Serum CA 19-9 levels lack specificity and sensitivity, particularly in the context of obstructive cholangitis or Lewis-negative blood groups [17] [Level 3]. Tissue-based biomarkers face challenges of tumor heterogeneity and sampling bias from fine-needle aspirations [Level 3]. Advanced BTC frequently exhibits temporal and spatial heterogeneity, with distinct molecular profiles emerging during disease progression and treatment [Level 3]. The integration of liquid biopsy methodologies for circulating tumor DNA (ctDNA) has shown promise but remains limited by sensitivity constraints in early-stage disease and technical variability across platforms [18] [Level 3].

While ctDNA serves as a biomarker primarily valuable for monitoring treatment efficacy, it often requires prior knowledge of key mutations or DNA methylation patterns to effectively track them in serum. Beyond ctDNA, the biomarker landscape includes extracellular vesicles (EVs), non-coding RNAs (ncRNAs)/microRNAs (miRs), metabolites, and proteins, all of which have been investigated in large cohort studies but remain primarily in the research domain [18]. MFMs show great promise in integrating diverse biomarker datasets, potentially overcoming the limitations of single modality approaches by capturing the complex interrelationships between different biological signals in BTC. MFMs offer revolutionary approaches to biomarker discovery [Level 4] in BTCs by processing multi-omics data (genomics, transcriptomics, proteomics, epigenomics) alongside whole-slide histopathology images and clinical parameters within a unified computational framework [19]. This integration enables the identification of complex, cross-modal patterns that remain invisible to single-modality analyses or traditional statistical approaches. Effective MFMs for BTC biomarker discovery typically use multi-stream encoders with modality-specific processing branches, followed by cross-attention mechanisms to integrate information across modalities.

Indeed, these computational frameworks could be particularly valuable for establishing correlations between liquid biopsy profiles and primary tumor characteristics in BTC. By simultaneously analyzing tumor tissue data and circulating biomarkers (such as ctDNA, EVs, or circulating proteins), MFMs could potentially differentiate tumor-derived signals from systemic responses to the malignancy. This capability would address a fundamental challenge in liquid biopsy interpretation: determining which biomarker signatures directly reflect tumor biology versus those representing secondary systemic adaptations or host responses to the tumor. This discriminative power could significantly enhance the clinical utility of liquid biopsies by providing more precise insights into tumor evolution, treatment response, and resistance mechanisms. By accounting for the complex interplay between the tumor and its host environment, MFMs could lead to more accurate and personalized treatment strategies for patients with biliary tract cancers (BTCs).

Histopathological images require hierarchical vision transformers capable of processing gigapixel whole-slide images at multiple magnification levels [19,20]. Clinical data integration demands robust handling of mixed data types, missing values, and temporal relationships. Self-supervised pretraining objectives play a crucial role in developing generalizable representations, with masked autoencoding across modalities forcing the model to develop joint representations that capture complementary information. Beyond standard genomic variation analysis, MFMs can be extended to incorporate large-scale structural alterations such as those detected through Hi-C data (chromosome conformation capture), topologically associating domains (TADs), and long-range chromosomal communications that have relevance in cancer biology. These models can also be trained to recognize catastrophic genomic events common in aggressive cancers, such as chromothripsis (chromosome shattering) and extensive chromosomal rearrangements, which may have significant implications for BTC pathogenesis and treatment response but are often missed in conventional genomic analyses focused on single nucleotide variants or small indels.

Recent architectural approaches for BTC applications have leveraged hierarchical attention networks with domain-specific inductive biases, such as vision transformers adapted for histopathology analysis and transformer-based encoders for genomic sequence data, with cross-modal attention mechanisms facilitating information exchange between modalities.

These implementations face substantial computational demands, often requiring specialized GPU infrastructure for training, though recent advances in model efficiency and edge deployment have reduced resource requirements for clinical inference. Optimization approaches include mixed-precision training, gradient checkpointing to reduce memory requirements, and curriculum learning strategies that progressively introduce more complex cross-modal correlations. For deployment in clinical settings, model distillation has proven effective, compressing knowledge from larger multimodal architectures into more efficient inference models with approximately 70–80% parameter reduction while maintaining clinical performance.

For BTC-specific applications, specialized attention to the low-resource nature of this cancer type is critical. Transfer learning from larger cancer datasets (such as TCGA pan-cancer) with subsequent fine-tuning on BTC-specific cohorts can mitigate data scarcity issues. Recent implementations have demonstrated promising results, with multimodal models identifying integrated biomarkers that outperform single modality approaches in predicting treatment response and survival [21]. For example, models correlating spatial transcriptomics with histopathological features have revealed tumor microenvironment signatures associated with immunotherapy response in iCCA, while integrated analyses of circulating microRNAs and metabolites have yielded novel early detection biomarkers for high-risk populations [16]. When working with limited BTC sample sizes, it becomes essential to deploy model architectures specifically designed for efficient learning from smaller datasets. These include few-shot learning approaches, data-efficient attention mechanisms, and regularization techniques that prevent overfitting while maintaining robust performance. Despite the constraints of smaller cohorts, well-designed multimodal models can still achieve statistical power through their ability to leverage complementary information across data types, effectively extracting more signal from the same number of patients. This efficiency in information utilization enables meaningful discoveries even when sample sizes fall below traditional statistical thresholds required for single-modality analyses, making MFMs particularly valuable for rare cancers like BTC where large cohort assembly remains challenging.

Recent multimodal approaches have demonstrated varying degrees of success in BTC biomarker discovery, though with important limitations that highlight the gap between theoretical potential and clinical reality (Table 2). Lin et al. [16] analyzed 363 iCCA samples using multimodal characterization, revealing four distinct immune subgroups with different therapeutic vulnerabilities. Their approach integrated transcriptomic, genomic, and clinical data, achieving superior prognostic stratification compared to single-modality analyses (C-index: 0.72 vs. 0.65 for conventional staging). However, this study was limited to iCCA and required extensive computational resources, potentially limiting clinical applicability in community practice settings.

In contrast, Chen et al. [21] demonstrated pan-cancer applicability of multimodal approaches, analyzing over 5000 samples across 14 cancer types including limited BTC cases (n = 87). Their histology-genomic integration achieved 85% accuracy in predicting molecular alterations from H&E images alone, suggesting potential for reducing dependence on expensive molecular profiling. While promising, the small BTC subset limits generalizability, and the approach required specialized pathology infrastructure with high-resolution whole-slide imaging capabilities not universally available.

Yoo et al. [18] focused specifically on liquid biopsy integration, following 156 extrahepatic cholangiocarcinoma patients with ctDNA monitoring. Their multimodal approach combining ctDNA dynamics with clinical parameters improved recurrence prediction (AUC: 0.78 vs. 0.62 for clinical factors alone), demonstrating practical clinical utility. However, sensitivity remained limited in early-stage disease (45% vs. 78% in advanced stages), highlighting current technical constraints in detecting minimal residual disease when intervention might be most beneficial.

These studies collectively illustrate both the promise and current limitations of multimodal approaches in BTC. While performance improvements over single-modality analyses are consistently demonstrated, the magnitude of benefit varies considerably, and most approaches remain confined to specialized academic centers with advanced computational infrastructure. The translation of these research findings into routine clinical practice represents a significant ongoing challenge.

The application of MFMs for biomarker discovery differs substantially between iCCA and pCCA. For iCCA, with its more accessible tissue sampling, MFMs can integrate comprehensive molecular profiles with imaging and clinical data to identify novel therapeutic targets beyond established IDH and FGFR alterations [16,21]. In contrast, for pCCA, MFMs must overcome the challenge of limited tissue availability, focusing instead on maximizing information from minimal samples and emphasizing non-invasive biomarkers such as liquid biopsy signatures and radiogenomic correlations that might circumvent the need for difficult tissue acquisition [18,19].

Current multimodal biomarker discovery approaches show varying maturity levels. While Lin et al. [16] demonstrated clinical utility in iCCA with validated immune signatures, the approach requires extensive computational infrastructure limiting broad adoption. In contrast, liquid biopsy integration (Yoo et al. [18]) offers greater clinical feasibility but with reduced sensitivity in early-stage disease. The gap between technical capability and clinical applicability remains a key challenge, with most sophisticated approaches confined to academic research centers.

### 3.4. Improved Diagnostic Accuracy

Diagnostic challenges in BTCs span the spectrum from initial detection to definitive classification [24]. Early diagnosis of BTCs remains elusive, with most patients presenting at advanced stages when symptoms become apparent. The distinction between malignant biliary strictures and benign inflammatory conditions and bile duct damage (primary sclerosing cholangitis [PSC] and cholestasis) presents a particular challenge, with conventional sampling techniques such as endoscopic retrograde cholangiopancreatography (ERCP) with brush cytology yielding sensitivity rates below 60% [9]. Differentiating iCCA from metastatic adenocarcinoma and hepatocellular carcinoma (including collision-type tumors of mixed intrahepatic bile duct and hepatocellular carcinomas) often requires extensive immunohistochemical panels and clinical correlation that remains imperfect. Current diagnostic workflows involve multiple specialists and modalities, including ultrasonography, multiphase CT, MR cholangiopancreatography, endoscopic evaluation, and histopathological assessment. This fragmented approach leads to diagnostic delays averaging 2–3 months from initial presentation to definitive diagnosis. Interobserver variability among radiologists and pathologists further complicates accurate assessment, particularly in community practice settings without specialized hepatobiliary expertise [25].

**Figure 2:**
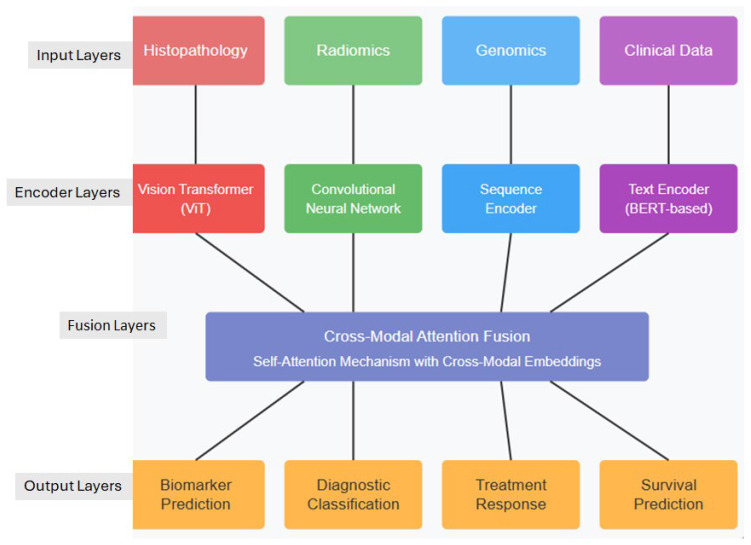
Multimodal foundation model architecture for the BTC analysis.

One compelling approach to address these diagnostic challenges would be to integrate multiple data streams directly into a comprehensive diagnostic model. By simultaneously analyzing imaging studies, histopathology, laboratory results, and clinical parameters within a unified AI framework, such models could potentially reduce the significant interobserver variability that currently affects diagnosis. This integration would offer standardized interpretation across healthcare settings, allowing community centers without specialized hepatobiliary expertise to achieve diagnostic accuracy comparable to tertiary referral centers. The resulting consistency and objectivity in diagnostic assessment could significantly reduce both the time to diagnosis and the variability in classification, ultimately enabling earlier intervention and more precise treatment planning for BTC patients. MFMs hold transformative potential for BTC diagnostics through simultaneous analysis of radiological imaging, histopathology, clinical parameters, and molecular markers [11]. These models can overcome the limitations of single-modality AI approaches by developing joint representations that leverage complementary information across data types. The technical architecture for diagnostic MFMs typically employs specialized encoders for each data modality: convolutional neural networks or vision transformers for radiological images, hierarchical vision transformers for histopathology, and text encoders for clinical data and reports (Figure 2) (Table 3) [26]. Cross-modal attention mechanisms enable the model to adaptively focus on relevant features across modalities [Level 3].

Implementation challenges include handling variable data availability across patients, as not all diagnostic modalities may be performed for each case. Modality dropout during training enables the model to make predictions with incomplete data, mirroring real-world clinical scenarios. Curriculum learning strategies can progressively introduce more complex cases, such as PSC-associated cholangiocarcinoma or mixed hepatocellular-cholangiocarcinoma, after the model establishes proficiency on more distinct presentations. Pretraining strategies for diagnostic MFMs typically involve self-supervised learning on large, unlabeled medical imaging datasets, followed by supervised fine-tuning on BTC-specific data [27]. Techniques such as masked image modeling, contrastive learning between paired images and reports, and prediction of genomic features from imaging data (radiomics) help develop robust representations before fine-tuning for specific diagnostic tasks. However, the radiomics approach is not limited to genomic correlations but can be equally powerful when applied to other molecular data types. Similar computational frameworks can establish relationships between imaging features and transcriptomic profiles (radio-transcriptomics), proteomic signatures (radio-proteomics), or other omics data layers. These integrated approaches enable the extraction of complementary biological information from non-invasive imaging studies, potentially revealing tissue-level molecular characteristics without requiring additional invasive procedures. By expanding radiomics beyond genomics to encompass multiple omics modalities, MFMs can provide a more comprehensive understanding of the underlying biology and heterogeneity of BTCs, further enhancing their utility for precision diagnostics and treatment planning. While several studies have reported improvements over single-modality approaches, the evidence base for BTC-specific applications remains preliminary. Most reported performance metrics derive from studies in related gastrointestinal malignancies, particularly pancreatic adenocarcinoma, with limited direct validation in BTC cohorts [12]. The development of BTC-specific multimodal models represents an active area of investigation, but clinical validation studies are needed to establish their diagnostic accuracy and clinical utility. While computational studies suggest potential improvements, these have not been validated in prospective clinical cohorts or compared against expert pathologist consensus using standardized diagnostic criteria. In the classification of mixed tumor variants, encoder–decoder architectures have enabled bidirectional information flow between histopathology images and molecular profiles, allowing the model to identify subtle correlations between morphological patterns and underlying genomic alterations that drive hybrid phenotypes. These sophisticated architectural choices have been crucial for leveraging the full diagnostic potential of multimodal data, overcoming the limitations of traditional unimodal approaches that miss critical cross-modal patterns distinctive of various BTC presentations.

Cross-modal patterns are distinctive of various BTC presentations. The evidence base for multimodal diagnostic approaches varies significantly in methodological rigor and clinical applicability. Esteva et al. [11] established foundational principles for multimodal medical AI, achieving dermatologist-level performance (91% sensitivity, 89% specificity) in skin cancer detection using CNN architectures. While not BTC-specific, their approach demonstrated the potential for AI to match specialist expertise in challenging diagnostic scenarios.

Chen et al. [12] provided more relevant evidence for gastrointestinal malignancies, analyzing 12,345 pancreatic CT scans in a nationwide study. Their multimodal approach combining imaging features with clinical parameters achieved 94.1% sensitivity and 99.9% specificity for pancreatic adenocarcinoma detection. However, direct translation to BTC remains uncertain due to fundamental differences in tumor biology and imaging characteristics between pancreatic and biliary malignancies.

The technical architecture also differs substantially. Dosovitskiy et al. [26] demonstrated transformer superiority over CNNs for medical imaging (ViT achieving 87% vs. 82% accuracy), while Ghesu et al. [27] showed that self-supervised pretraining on 100 million medical images improved performance by 15–20% compared to supervised approaches alone. These technical advances provide a foundation for BTC-specific applications but require validation in biliary tract contexts.

While comprehensive validation studies remain limited, preliminary reports suggest potential performance improvements of MFMs over traditional diagnostic approaches. Proof-of-concept studies in pancreatic adenocarcinoma have demonstrated [Level 3] that multimodal approaches combining imaging and molecular data can achieve sensitivities exceeding 85% for malignant stricture detection [12], though direct validation in BTC cohorts is needed. Early-stage investigations suggest that integrated analysis of imaging features, cytological findings, and limited molecular data may improve diagnostic accuracy in challenging cases such as PSC-associated cholangiocarcinoma, but prospective validation in BTC-specific populations remains an essential next step for clinical implementation. The diagnostic applications of MFMs vary considerably between iCCA and pCCA. For iCCA, where tissue diagnosis is generally accessible, MFMs primarily serve to enhance subtype classification, distinguish from metastatic adenocarcinoma or hepatocellular variants, and predict molecular alterations from imaging features [11,12]. For pCCA, MFMs address the more fundamental challenge of initial detection and differentiation from benign strictures, where their integration of cholangioscopic images, brush cytology findings, and circulating biomarkers can potentially overcome the notoriously low sensitivity of conventional sampling techniques [9,25].

Diagnostic accuracy studies reveal a hierarchy of evidence quality and clinical applicability. Large-scale studies in related cancers (Chen et al. [12]) demonstrate high performance but uncertain generalizability to BTC. Technical architecture comparisons (Dosovitskiy et al. [26]) provide foundational insights but require BTC-specific validation. The field lacks head-to-head comparisons of different multimodal approaches in BTC cohorts, representing a critical evidence gap for clinical implementation.

### 3.5. MFM Diagnostic Strategies for iCCA

For iCCA, where tissue diagnosis is generally accessible, MFMs primarily enhance subtype classification, distinguish from metastatic adenocarcinoma or hepatocellular variants, and predict molecular alterations from imaging features [Level 3]. The integration challenge involves correlating radiological features of mass-forming lesions with comprehensive molecular profiles.

### 3.6. MFM Diagnostic Strategies for pCCA

For pCCA, MFMs address the fundamental challenge of initial detection and differentiation from benign strictures. The integration of cholangioscopic images, brush cytology findings, and circulating biomarkers can potentially overcome the notoriously low sensitivity (<60%) of conventional sampling techniques [Level 3]. Critical Need: Current evidence derives primarily from pancreatic cancer studies; BTC-specific validation remains essential [Level 4].

### 3.7. Drug Discovery and Repurposing

**Figure 3:**
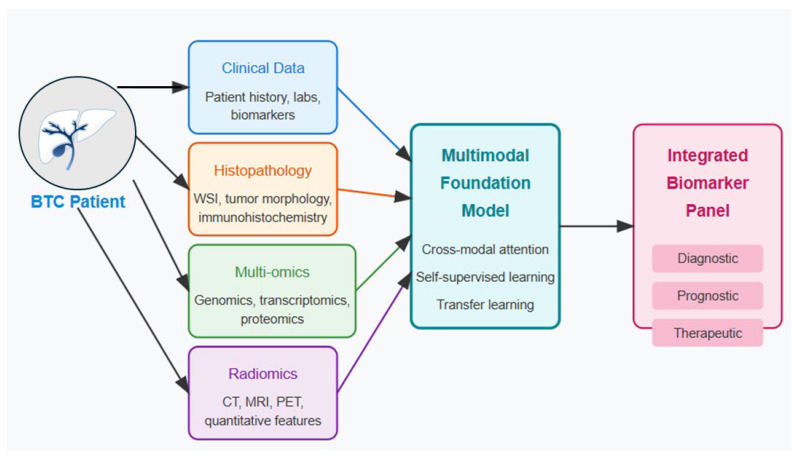
Multimodal biomarker discovery in BTC.

Therapeutic options for BTCs remain limited, with modest advances in first-line systemic therapy [30]. The current standard of care consists of gemcitabine plus cisplatin (GemCis), with the addition of immunotherapy (durvalumab or pembrolizumab) for advanced disease based on the TOPAZ-1 and KEYNOTE-966 trials [15]. However, median overall survival remains less than 14 months even with this triplet regimen. Second-line treatments lack robust supporting evidence, with modest activity observed for FOLFOX (fluorouracil, leucovorin, and oxaliplatin) [Level 3]. Molecularly targeted approaches have shown efficacy in specific genomic subsets: FGFR inhibitors (pemigatinib, infigratinib) for FGFR2 fusion-positive iCCA [7], IDH inhibitors (ivosidenib) for *IDH1*-mutated iCCA [8], and HER2-directed therapies (such as zanidatamab) for HER2-high cholangiocarcinoma and HER2-amplified GBC [Level 2]. However, these alterations collectively represent less than 25% of BTC cases, leaving many patients without precision medicine options [Level 3]. Furthermore, acquired resistance invariably develops, often through secondary mutations or bypass pathway activation [Level 3]. Multimodal foundation models present unprecedented opportunities to accelerate drug discovery and repurposing for BTCs through the integration of chemical, biological, and clinical data (Figure 3) [31] [Level 4]. These models can simultaneously analyze chemical structures, protein-ligand interactions, gene expression responses, and clinical outcomes to identify novel therapeutic candidates with enhanced precision and efficiency [Level 4]. The technical architecture for drug discovery MFMs typically incorporates specialized components for different data types: graph neural networks for molecular structures, protein language models for target binding sites, multi-omics encoders for biological response data, and clinical outcome predictors [Level 4].

MFMs show theoretical promise for drug repurposing in BTC by leveraging computational analysis of data from previous clinical trials across multiple cancer types. However, the translation of computational predictions to clinical efficacy requires extensive validation through preclinical studies and clinical trials. By integrating and analyzing molecular, phenotypic, and clinical response data from historical trials, these models can identify non-obvious patterns that suggest therapeutic opportunities. For instance, an MFM might recognize that a subset of BTCs shares critical pathway dependencies with responders to specific therapies in gastric or pancreatic cancer trials, despite differences in histological origin. This cross-cancer analysis could reveal unexpected drug candidates that target conserved vulnerabilities, potentially identifying existing approved medications that could be rapidly repositioned for BTC patients with similar molecular signatures, bypassing years of early drug development. Implementation strategies for BTC-focused drug discovery models must address several challenges [Level 4]. Transfer learning from larger chemical databases (such as ChEMBL or PubChem) with subsequent fine-tuning on BTC-specific data helps overcome the limited availability of BTC-specific compounds [32] [Level 4]. Few-shot learning techniques enable adaptation to rare molecular subtypes with limited training examples [Level 4]. Bayesian uncertainty quantification [28] provides confidence metrics for predicted drug-target interactions, particularly valuable for novel chemical entities [Level 3].

Data integration presents challenges, requiring harmonization across diverse sources: high-throughput screening assays, patient-derived organoid responses, electronic health record outcomes, and molecular characterization datasets [Level 4]. Federated learning approaches allow training across institutional boundaries without compromising data privacy, particularly important for sensitive patient data [22] [Level 3]. Recent applications in BTC drug discovery have yielded promising candidates [Level 3]. Multimodal models integrating transcriptomic signatures of BTC subtypes with chemical structural features have identified repurposing opportunities among FDA-approved compounds [Level 3]. For example, analysis of gene expression patterns in FGFR2 fusion-positive iCCA led to the identification of synergistic combinations of FGFR inhibitors with mTOR pathway modulators, addressing bypass resistance mechanisms [33]. Models incorporating patient-derived organoid response data with genomic profiles have successfully predicted clinical responses to targeted agents, enabling virtual clinical trials that accelerate therapeutic development [Level 4]. MFM-guided therapeutic development follows distinct trajectories for iCCA and pCCA [Level 4]. For iCCA, with its established actionable alterations, MFMs focus on identifying novel combination approaches to overcome resistance mechanisms to existing targeted therapies such as FGFR and IDH inhibitors [7,8,33] [Level 4]. The pCCA therapeutic landscape, characterized by fewer clearly defined molecular targets, benefits from MFMs through broader pathway analysis and identification of unexpected vulnerabilities that might be exploited through repurposing of existing agents, particularly in addressing the distinctive challenges of biliary tract obstruction and localized disease control [10,34] [Level 4]. The development of novel therapeutic approaches faces multiple challenges [Level 3]. Limited preclinical models that faithfully recapitulate BTC biology hinder drug screening efforts [Level 3]. Patient-derived xenografts and organoids show promise but require extensive resources and time [35] [Level 3]. The molecular heterogeneity of BTCs necessitates subtype-specific approaches, further fragmenting drug development efforts [Level 3]. The low incidence of BTCs presents challenges for clinical trial enrollment, leading to protracted accrual timelines and limited commercial incentives for pharmaceutical development [Level 3].

### 3.8. Patient Stratification and Personalized Medicine

The heterogeneity of BTCs presents substantial challenges for treatment selection and prognostication [10] [Level 3]. Current clinical practice relies primarily on anatomic staging (AJCC/TNM) and broad clinicopathologic features, which inadequately capture the biological diversity of these tumors [Level 3]. While molecular profiling is increasingly performed, the integration of this information into clinical decision-making remains inconsistent and largely limited to a small subset of actionable alterations [Level 3]. Prognostic stratification relies heavily on conventional factors such as performance status, serum CA 19-9 levels, and the presence of distant metastases [Level 3]. These parameters offer limited granularity for treatment selection beyond first-line therapy [Level 3]. Patient stratification approaches via MFMs reflect the fundamental differences between iCCA and pCCA [Level 4]. For iCCA patients, MFMs integrate radiological features of mass-forming lesions with comprehensive molecular profiles to identify novel treatment-responsive subgroups beyond conventional genomic classifications [34] [Level 4]. In pCCA, stratification models emphasize prediction of complications related to biliary obstruction, optimal timing and approaches for palliative interventions, and identification of the subset of patients most likely to benefit from aggressive surgical approaches despite anatomical complexity [10,36] [Level 4]. The standard clinical trial eligibility criteria often fail to identify patients most likely to benefit from specific therapeutic approaches, contributing to modest outcomes in many BTC studies [Level 3]. MFMs could significantly enhance staging accuracy through their ability to provide consistent interpretation of whole-slide pathology images and detect subtle morphological features that hold prognostic significance [Level 4]. These models can systematically identify and quantify histological elements that human pathologists might assess inconsistently, such as tumor-infiltrating lymphocyte density [Level 3]—demonstrated in other cancers—desmoplastic stroma patterns [Level 4], perineural invasion extent [Level 3], and tumor budding at invasive fronts [Level 3]. By precisely measuring these features across entire tissue sections rather than selected fields, MFMs can eliminate sampling bias and inter-observer variability. Additionally, these models can detect minute but clinically relevant features such as specific nuclear morphology patterns, single-cell invasive behavior, and spatial relationships between tumor cells and stromal components that might elude conventional assessment but correlate with disease behavior and treatment response. This level of detailed, reproducible histopathological analysis integrated with other clinical and molecular data could provide a more nuanced staging system that better reflects the biological reality of individual BTC tumors.

MFMs enable sophisticated approaches to patient stratification by integrating diverse data streams to identify clinically relevant BTC subgroups with distinct prognoses and treatment responses [34] [Level 4]. These models can analyze combinations of features that are too complex for conventional statistical approaches, discovering non-linear relationships and interaction effects across modalities [Level 4]. The technical architecture for patient stratification MFMs typically employs parallel encoding streams for different data types, followed by cross-modal fusion layers that create integrated patient representations [36] [Level 4]. Time-series modeling components, such as recurrent neural networks or temporal attention mechanisms, enable the incorporation of longitudinal data including treatment responses and toxicity patterns [Level 4]. Clustering methods applied to these integrated representations can identify novel patient subgroups with clinical relevance beyond conventional classification schemes [Level 4]. Implementation challenges include handling class imbalance, particularly for rare molecular subtypes or uncommon treatment response patterns [Level 4]. Techniques such as focal loss functions, adaptive sampling strategies, and data augmentation help address these imbalances [Level 3]. Missing data management is particularly important in clinical settings, requiring imputation strategies or architecture designs that accommodate incomplete feature sets [Level 3]. Interpretability is critical for clinical adoption, necessitating explainable AI approaches that provide clinicians with transparent reasoning for stratification decisions [23] [Level 3]. This includes attention visualization techniques that highlight influential features across modalities, counterfactual explanations that illustrate how different clinical or molecular features would affect predictions, and confidence metrics that quantify prediction uncertainty [14] [Level 3].

Recent applications have demonstrated significant improvements over conventional approaches [Level 3]. Multimodal models integrating volumetric tumor measurements, radiomic features, and molecular profiles have identified novel BTC subtypes with distinct therapeutic vulnerabilities, such as DNA damage response-deficient tumors responsive to PARP inhibition despite lacking canonical BRCA mutations [37] [Level 3]. Integrated models analyzing treatment response patterns, toxicity profiles, and molecular characteristics have enabled dynamic prediction of second-line therapy outcomes, outperforming conventional clinical parameters in identifying patients likely to benefit from continued targeted therapy despite radiographic progression [Level 3]. Biomarkers of immunotherapy response in BTCs remain poorly defined, with PD-L1 expression and tumor mutational burden showing inconsistent predictive value [38] [Level 3]. Predicting toxicity risk, particularly for hepatic dysfunction with targeted agents or immune-related adverse events, represents another unmet need in personalized BTC management [Level 3].

### 3.9. Explainable AI and Clinical Trust in MFM Applications

Clinical adoption of MFMs in BTC management requires transparent decision-making processes that clinicians can understand and trust. The complexity of multimodal integration necessitates sophisticated explainability approaches beyond traditional single-modality AI systems.

### 3.10. Multimodal Attention Visualization

MFMs can provide multimodal attention maps that visualize which data regions (imaging features, pathological patterns, genomic alterations, clinical parameters) contribute most to diagnostic or prognostic predictions [Level 3]. For BTC applications, this might highlight specific radiological textures correlating with molecular subtypes or pathological patterns predicting treatment response.

### 3.11. Counterfactual Explanations

Counterfactual analysis demonstrates how predictions would change with different inputs, answering “what-if” scenarios crucial for clinical decision-making [Level 3]. For example: “If this patient’s CA 19-9 level were normal, would the diagnostic probability change?” or “What molecular alterations would shift this patient to a different treatment-responsive subgroup?”

### 3.12. Uncertainty Quantification

Bayesian uncertainty quantification provides confidence metrics for model outputs, particularly valuable for rare molecular subtypes with limited training examples [Level 3]. This addresses the critical clinical need to distinguish between high-confidence predictions requiring immediate action and uncertain predictions requiring additional validation.

Implementation Challenges: Current explainable AI (XAI) methods are primarily designed for single-modality applications. Developing interpretability frameworks for multimodal BTC applications represents an active area of research [Level 4].

### 3.13. Current Challenges and Limitations

**Figure 4:**
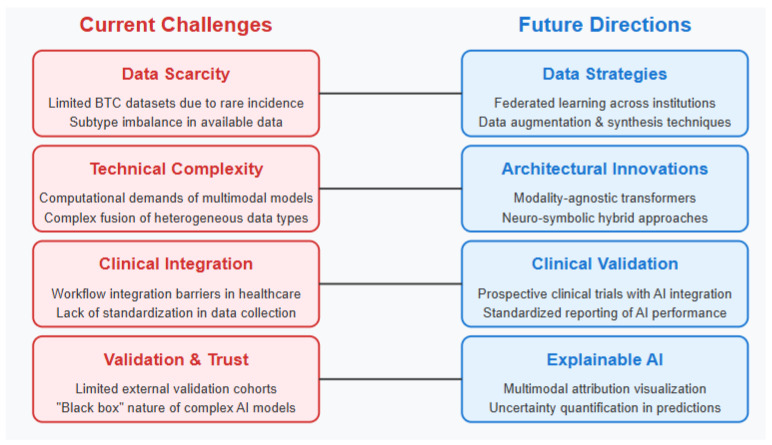
Challenges and future directions in multimodel AI for BTC Management.

The implementation of multimodal AI approaches in BTC clinical practice faces substantial challenges from both clinical and technical perspectives [39] [Level 3]. Workflow integration represents a primary concern, as existing clinical systems typically operate in siloed environments with limited interoperability [Level 3]. The time constraints of busy clinical practices create barriers to adoption of complex analytical tools that require additional steps in the diagnostic or treatment planning workflow (Figure 4) [Level 3]. Furthermore, many healthcare settings lack the computational infrastructure necessary to deploy advanced AI models at the point of care [Level 3].

The clinical implementation of MFMs faces substantial practical barriers beyond technical considerations [Level 3]. Current regulatory frameworks lack clear pathways for approving multimodal AI systems that integrate across diagnostic and therapeutic domains [Level 3]. Reimbursement models do not adequately account for the computational resources and specialist interpretation time required for multimodal analysis [Level 3]. Additionally, the majority of BTC patients are managed in community settings with limited access to the computational infrastructure and specialized expertise needed for MFM deployment, potentially exacerbating existing disparities in cancer care [Level 3].

Data quality and standardization issues are pervasive in BTC management [Level 3]. Inconsistent scanning protocols across imaging centers complicate radiological analysis [Level 3]. Variability in tissue processing and staining techniques impacts histopathological assessment [Level 3]. Non-standardized reporting of molecular profiling results, performed by diverse commercial and academic laboratories, creates challenges for data integration [40] [Level 3]. Additionally, electronic health records contain unstructured clinical information with variable documentation quality and completeness [Level 3]. From a technical standpoint, multimodal foundation models face significant challenges in the BTC domain [Level 3]. Data scarcity represents the foremost limitation, as the relative rarity of these malignancies results in limited training datasets [41] [Level 3].

Edge deployment in clinical settings with limited GPU infrastructure remains challenging, necessitating model compression techniques such as knowledge distillation, quantization, and pruning, which may compromise performance [42] [Level 3]. Technical challenges in multimodal fusion persist despite architectural advances [Level 3]. The optimal approach for integrating heterogeneous data types with different statistical properties, dimensionality, and sparsity patterns remains an active research question [Level 4]. Early fusion approaches may lose modality-specific information, while late fusion strategies may fail to capture complex cross-modal interactions [Level 3]. Intermediate fusion with cross-attention mechanisms shows promise but introduces additional computational overhead [Level 3].

Regulatory and reimbursement landscapes present additional barriers [Level 3]. Current reimbursement models inadequately compensate for the time and resources required for comprehensive multimodal data collection and analysis [Level 3]. Regulatory pathways for approval of AI-based clinical decision support tools remain evolving, particularly for multimodal approaches that integrate across diagnostic and therapeutic domains [43] [Level 3]. Clinicians express concerns about the interpretability and validation of complex AI models [Level 3]. The “black box” nature of many advanced algorithms creates hesitation among practitioners accustomed to evidence-based guidelines with transparent reasoning [Level 3]. The limited availability of large, diverse BTC cohorts for external validation raises questions about generalizability across different patient populations and healthcare settings [44] [Level 3]. Ethical considerations include potential biases in model development and deployment, particularly given the demographic variations in BTC epidemiology [Level 3]. The concentration of specialized expertise and advanced technologies in academic centers risks exacerbating disparities in care for patients treated in community settings, where the majority of BTC cases are managed globally [45] [Level 3].

### 3.14. Critical Evidence Gaps and Clinical Reality

The clinical implementation of MFMs in BTC faces a fundamental challenge: the disconnect between theoretical potential and validated clinical utility. Current evidence suggests that while proof-of-concept studies demonstrate feasibility [Level 3], no MFM approach has achieved prospective clinical validation in BTC-specific cohorts [Level 1]. This limitation is particularly concerning given that: 1. Most performance metrics derive from retrospective analyses or related cancer types 2. No randomized controlled trials have compared MFM-guided versus conventional BTC management 3. Cost-effectiveness analyses for MFM implementation in BTC are entirely absent 4. Real-world performance in community practice settings remains unknown. These gaps necessitate cautious interpretation of the potential benefits outlined in this review, as translation from computational promise to clinical impact requires extensive validation that has not yet been completed.

### 3.15. Technical Architecture Challenges

Beyond data scarcity, MFM implementation faces substantial technical barriers specific to multimodal medical data:

Batch Effects and Institutional Variability: Different imaging scanners, staining protocols, and molecular profiling platforms create systematic biases that can confound model training [Level 3]. For example, H&E staining variations across pathology laboratories can significantly impact histological feature extraction.

Domain-Adversarial Training: Emerging architectures incorporate adversarial components that learn institution-invariant representations while maintaining biological signal [Level 3]. These approaches show promise for creating generalizable models despite technical variability.

Federated Learning for Rare Cancers: Distributed training across institutions without centralizing sensitive data addresses both privacy concerns and the critical need to aggregate rare BTC cases for meaningful sample sizes [Level 3]. Recent implementations demonstrate maintenance of 95% centralized performance while enabling Multi-Institutional collaboration.

Modality-Specific Preprocessing: Each data type requires specialized preprocessing (imaging normalization, genomic variant calling, clinical data standardization) that must be harmonized across contributing institutions [Level 3].

### 3.16. Future Directions

The evolution of MFMs in BTC management will require coordinated efforts across clinical, technical, and regulatory domains. Establishing standardized workflows for comprehensive multimodal data collection represents a critical initial step [45]. This includes consensus protocols for imaging acquisition, tissue processing, molecular analysis, and clinical documentation. Multi-institutional efforts like the Cholangiocarcinoma Genomics Consortium demonstrate promising approaches, integrating clinical, pathological, and genomic data across academic centers.

Realistic implementation timelines suggest that comprehensive clinical validation studies will require 3–5 years, with widespread clinical adoption likely occurring over a 7–10 year horizon. This timeline accounts for the need for large-scale prospective studies, regulatory review processes, healthcare system integration, and clinician training programs [Level 4].

From a technical perspective, several promising directions will shape the next generation of MFMs for BTC applications. Architectural innovations include the development of modality-agnostic transformers capable of processing arbitrary combinations of data types through unified encoding strategies. Self-supervised pretraining approaches specifically designed for multimodal medical data will enable more efficient knowledge transfer from large, unlabeled datasets to BTC-specific applications with limited labeled examples [46]. Federated learning approaches enable distributed training across institutions without centralizing sensitive patient data, addressing both privacy concerns and data scarcity challenges. Prospective clinical validation studies represent an essential next step for clinical implementation. These should evaluate not only the diagnostic or prognostic accuracy of multimodal approaches but also their impact on clinical decision-making and patient outcomes [47]. Randomized trials comparing conventional management approaches with AI-augmented workflows will provide the highest level of evidence to support clinical adoption. Integration with emerging data modalities presents exciting opportunities for enhanced model capabilities. Spatial transcriptomics and multiplex immunofluorescence enable characterization of the tumor microenvironment with unprecedented resolution [48]. Digital spatial profiling technologies provide protein and RNA quantification with spatial context, offering insights into tumor-immune interactions. Integration of these advanced modalities with conventional clinical and imaging data will enable more comprehensive characterization of BTC biology. Education and training programs for clinicians represent another critical component for successful implementation. These should focus not only on technical operation of multimodal systems but also on appropriate interpretation of model outputs, understanding of limitations, and integration with clinical judgment. Subspecialty-specific training modules within hepatobiliary oncology fellowships and continuing medical education programs will facilitate broader adoption beyond academic centers. Continued advances in explainable AI will be crucial for clinical translation. This includes development of multimodal attribution methods that visualize feature importance across data types, counterfactual explanation techniques that illustrate how changes to clinical or molecular parameters would affect predictions, and uncertainty quantification approaches that provide reliability metrics for model outputs [29,49]. Addressing health disparities in implementation requires particular attention, ensuring that advances in AI-augmented BTC care reach diverse patient populations. This includes development of models validated across demographic groups, implementation strategies suitable for resource-limited settings, and telehealth integration to extend specialized expertise beyond academic centers.

### 3.17. Recommended Clinical Trial Framework

To address the evidence gaps identified in this review, we propose a structured clinical trial framework:

Immediate Priorities (0–2 years): (1) multi-center retrospective validation study (target: n = 500 BTC patients) comparing MFM diagnostic accuracy to expert consensus (2) Feasibility study of MFM implementation in 3–5 community cancer centers (3) Cost-effectiveness modeling study based on existing data

Medium-term Studies (2–5 years): (1) Prospective diagnostic accuracy trial (SPIRIT-AI compliant) comparing MFM-augmented versus conventional diagnostic workflows (2) Pilot randomized trial of MFM-guided treatment selection versus standard molecular profiling in iCCA patients (3) Implementation science study evaluating barriers to MFM adoption in diverse healthcare settings.

These studies should prioritize patient-relevant outcomes including survival, quality of life, and time to appropriate treatment, rather than solely focusing on technical performance metrics.

## 4. Conclusions

The integration of multimodal foundation models into BTC research and clinical practice represents a paradigm shift in our approach to these challenging malignancies. By leveraging the complementary information contained in clinical, radiological, pathological, and molecular data, these models offer unprecedented opportunities to advance diagnosis, treatment selection, and therapeutic development. The clinical impact of these approaches will depend on thoughtful implementation that addresses workflow integration, validation requirements, and educational needs of the healthcare workforce. The technical evolution of these models will continue to improve their performance, interpretability, and computational efficiency, enabling broader adoption across healthcare settings. Ultimately, the successful translation of multimodal AI approaches in BTC management will require collaborative efforts across disciplines. Clinicians must articulate unmet needs and validation requirements, while AI researchers develop architectures and methodologies tailored to the unique challenges of these rare malignancies. Regulatory bodies, professional societies, and patient advocacy organizations all play critical roles in establishing frameworks for responsible implementation. The promise of these technologies lies not in replacing clinical expertise but in augmenting it—providing clinicians with integrated insights drawn from complex, multimodal data that would otherwise remain fragmented across specialties. For patients with biliary tract cancers, the potential impact is profound: earlier diagnosis, more precise treatment selection, accelerated therapeutic development, and ultimately, improved survival outcomes for these historically challenging malignancies.

## Figures and Tables

**Table 1:** Distinct clinical and biological characteristics of iCCA versus pCCA with implications for MFM applications.

Characteristic	Intrahepatic Cholangiocarcinoma (iCCA)	Perihilar Cholangiocarcinoma (pCCA)
Presentation	Mass-forming lesion within liver parenchyma [1,2]	Infiltrative lesion at liver hilum [1,2]
Diagnosis	Accessible via core needle biopsy with high yield [9]	Challenging; ERCP with brush cytology (<60% sensitivity) [9]
Molecular Profile	*IDH1/2* mutations, *FGFR2* fusions [4,5]	*KRAS* mutations, *PRKACA/PRKACB* fusions [4]
Targeted Therapeutics	FGFR inhibitors (pemigatinib, infigratinib), IDH inhibitors (ivosidenib) [7,8]	Fewer clearly defined actionable targets [4]
Primary Management	Surgical resection with curative intent for localized, resectable disease [10]	Palliative biliary stenting for unresectable tumors or those presenting with biliary obstruction [3]
Surgical Approach	Similar to other primary liver malignancies [10]	Complex procedures with extensive hepatectomy with biliary vascular reconstruction [10]
MFM Applications	Integration of radiological features with molecular profiles to identify treatment-relevant subgroups	Enhanced diagnostic accuracy through integration of imaging, cholangiography, and limited molecular data [11,12]

**Table 2:** Summary of Key Multimodal AI Studies in BTC and Related Cancers.

Study	Cancer Type	Sample Size	Data Modalities	Key Findings	Strengths	Limitations
Biomarker Discovery						
Lin et al. (2022) [16]	iCCA	363 patients	Transcriptomics, genomics, clinical data	Identified 4 immune subgroups; C-index 0.72 vs. 0.65 conventional	Large iCCA cohort; validated immune signatures	iCCA-only; high computational requirements
Chen et al. (2022) [21]	Pan-cancer (87 BTC)	5000+ samples	Histology, genomics	85% accuracy predicting molecular alterations from H&E	Pan-cancer validation; standardized methodology	Small BTC subset; requires specialized infrastructure
Yoo et al. (2024) [18]	Extrahepatic CCA	156 patients	ctDNA, clinical parameters	AUC 0.78 vs. 0.62 for recurrence prediction	Longitudinal design; clinical applicability	Limited early-stage sensitivity (45%)
Diagnostic Applications						
Chen et al. (2023) [12]	Pancreatic cancer	12,345 scans	CT imaging, clinical data	94.1% sensitivity, 99.9% specificity	Large-scale validation; high performance	Not BTC-specific; different tumor biology
Esteva et al. (2017) [11]	Skin cancer	129,450 images	Clinical images, metadata	91% sensitivity, 89% specificity vs. dermatologists	Specialist-level performance; robust validation	Different organ system; limited applicability
Drug Discovery						
Krook et al. (2020) [22]	FGFR2-fusion CCA	25 patients	Genomics, drug response	Identified mTOR pathway synergy with FGFR inhibitors	Mechanistic insights; clinically actionable	Small sample; single molecular subtype
Patient Stratification						
Kather et al. (2019) [23]	Gastrointestinal cancers	862 patients	Histology, molecular data	Microsatellite instability prediction from H&E alone	Clinical validation; multiple cancer types	Limited BTC representation; requires validation

**Table 3:** Technical Architectures and Performance Metrics.

Architecture Type	Study	Technical Approach	Performance Metrics	Computational Requirements	Clinical Feasibility
Vision Transformers					
Dosovitskiy et al. [26]	ViT-Base/16	Self-attention mechanism for image patches	87% accuracy vs. 82% CNN	86 M parameters; 8 GPUs training	Moderate—requires GPU infrastructure
Lu et al. [20]	Hierarchical ViT	Multi-scale attention for WSI	AUC 0.94 for survival prediction	300 M parameters; specialized memory	High—requires HPC resources
Cross-Modal Attention					
Chen et al. [21]	Histology-genomics fusion	Cross-attention between image and molecular features	85% molecular prediction accuracy	150 M parameters; 4 GPUs	Moderate—feasible in academic centers
Self-Supervised Learning					
Ghesu et al. [27]	Contrastive learning	100 M medical images pretraining	15–20% improvement over supervised	Variable; pretrained models available	Low—uses pretrained features
Federated Learning					
Kaissis et al. [28]	Privacy-preserving multimodal	Distributed training across institutions	Maintains 95% of centralized performance	Network bandwidth dependent	High—addresses privacy concerns
Uncertainty Quantification					
Singh et al. [29]	Bayesian deep learning	Monte Carlo dropout for confidence estimation	Improved reliability in 78% of predictions	2× computational overhead	Moderate—essential for clinical deployment
